# ISS mapped from ICD-9-CM by a novel freeware versus traditional coding: a comparative study

**DOI:** 10.1186/1757-7241-18-17

**Published:** 2010-03-31

**Authors:** Stefano Di Bartolomeo, Silvia Tillati, Francesca Valent, Loris Zanier, Fabio Barbone

**Affiliations:** 1 Agenzia Regionale della Sanità del Friuli Venezia Giulia, Udine, Italy; 2 Istituto di Igiene ed Epidemiologia, Azienda Ospedaliero-Universitaria di Udine, Udine, Italy; 3 Cattedra di Epidemiologia, Università degli Studi di Udine, Udine, Italy

## Abstract

**Background:**

Injury severity measures are based either on the Abbreviated Injury Scale (AIS) or the International Classification of diseases (ICD). The latter is more convenient because routinely collected by clinicians for administrative reasons. To exploit this advantage, a proprietary program that maps ICD-9-CM into AIS codes has been used for many years. Recently, a program called ICDPIC trauma and developed in the USA has become available free of charge for registered STATA^® ^users. We compared the ICDPIC calculated Injury Severity Score (ISS) with the one from direct, prospective AIS coding by expert trauma registrars (dAIS).

**Methods:**

The administrative records of the 289 major trauma cases admitted to the hospital of Udine-Italy from 1 July 2004 to 30 June 2005 and enrolled in the Italian Trauma Registry were retrieved and ICDPIC-ISS was calculated. The agreement between ICDPIC-ISS and dAIS-ISS was assessed by Cohen's Kappa and Bland-Altman charts. We then plotted the differences between the 2 scores against the ratio between the number of traumatic ICD-9-CM codes and the number of dAIS codes for each patient (DIARATIO). We also compared the absolute differences in ISS among 3 groups identified by DIARATIO. The discriminative power for survival of both scores was finally calculated by ROC curves.

**Results:**

The scores matched in 33/272 patients (12.1%, k 0.07) and, when categorized, in 80/272 (22.4%, k 0.09). The Bland-Altman average difference was 6.36 (limits: minus 22.0 to plus 34.7). ICDPIC-ISS of 75 was particularly unreliable. The differences increased (p < 0.01) as DIARATIO increased indicating incomplete administrative coding as a cause of the differences. The area under the curve of ICDPIC-ISS was lower (0.63 vs. 0.76, p = 0.02).

**Conclusions:**

Despite its great potential convenience, ICPIC-ISS agreed poorly with its conventionally calculated counterpart. Its discriminative power for survival was also significantly lower. Incomplete ICD-9-CM coding was a main cause of these findings. Because this quality of coding is standard in Italy and probably in other European countries, its effects on the performances of other trauma scores based on ICD administrative data deserve further research. Mapping ICD-9-CM code 862.8 to AIS of 6 is an overestimation.

## Introduction

When investigating trauma care, injury severity is an important variable to adjust for. The most common method for measuring injury severity is still the Injury Severity Score (ISS) [1] that is based on the Abbreviated Injury Scale (AIS) [2]. Other methods have been devised like ICISS [3] or TMPM-ICD9 [4] that are based instead on the international classification of diseases (ICD). These latter methods have been reported in the United States to outperform ISS in predictive models [5,6] and their use is expanding [4].

One main advantage of ICD-based scales is that they use information routinely collected by clinicians for administrative reasons, and no additional labour or expenses are required. Conversely, AIS coding requires specific expertise, dedicated time and the purchase of a proprietary manual. In case of multiple injuries, though, the administrative charts may be less complete if they allow the registration of a limited number of diagnoses (six in Italy) and within this number non traumatic diagnoses (e.g. medical complications) need also to be fitted. Another potential difference that might affect coding quality is that usually ICD codes in administrative files are assigned by busy physicians on duty, while the assignment of AIS codes is done by committed personnel.

In summary, on one hand AIS/ISS is still desirable for comparative injury collection worldwide, on the other hand practically everyone can use ICD. To try and exploit the advantages of both, a commercially available software that converts the codes from the ICD, Ninth Revision, Clinical Modification (ICD-9-CM) into AIS 1990/ISS scores has been available for many years (ICDMAP-90, Tryanalitics, Baltimore USA). Since April 2009, this function is offered also by a program (ICDPIC-trauma, hereafter called ICDPIC, by Clark DE, Osler TM, Hahn DR) that is downloadable free of charge from within the statistical package STATA, versions 8.0 or higher [7].

The objective of this paper is to compare the ISS calculated by ICDPIC from routinely collected administrative data with the one based on direct AIS coding by expert registrars.

## Materials and methods

We used the database of the Italian Trauma Registry (RITG) and the standard administrative database of hospital discharge records (ADB) of the region Friuli Venezia Giulia, Italy.

The RITG is a national database that was launched in 2004 [8] as a research project. Currently it comprises about 3000 cases and is used by 12 hospitals. The registry includes trauma cases with ISS>15 or admitted to an intensive care unit. Within this registry, we focused on the cases admitted to the hospital 'Azienda Ospedaliero-Universitaria' of Udine in the region Friuli Venezia Giulia. The cases were accrued from July 1 2004 to June 30 2005, when the hospital withdrew from the registry at the end of the pilot phase, and are the only patients of the region Friuli Venezia Giulia, for which AIS scores directly and prospectively assigned are available. According to the quality assurance protocol of the pilot phase of RITG, the AIS scores had been assigned by two experienced researchers using the 1990 revision, update 1998, and, in case of discrepancy, external judgment had been sought from the researchers of another participating hospital. In the case of multiple injuries, all injuries were coded and recorded. No ICD-9-CM diagnoses were recorded in RITG.

The ADB contains standardised information on all hospital admissions of the region. Up to six ICD-9-CM diagnostic codes are part of this information.

We selected from the ADB database all cases with a traumatic (800- 959.9) diagnostic code, and then we linked the 289 cases from RITG with the correspondent administrative records using a two-staged procedure. All matches for sex, age, date and ward of admission were initially identified, and then multiple matches and RITG cases with no match in the ADB were further assessed. To refine the linkage of these cases, we used personal data like name, surname or date of birth, which are stored separately in respect of the current legislation.

The ethical committee of the hospital had already approved RITG and the patients or relatives had signed a written consent for their data to be further used for quality-improvement purposes. As for the ADB, some of the authors are entitled to access sensitive data in the terms of the local legislation. Following the linkage between the two databases, the data were immediately re-anonimized and no sensitive data were present in the final database.

Using the statistical package Stata SE version 10 and the additional, freely downloadable, program ICDPIC, we calculated the ICD-9-CM derived ISS and compared it with the directly calculated ISS. The Cohen's kappa measure was used to assess the agreement of the two scores. Cohen's kappa was originally developed to examine inter-observer agreement on diagnostic tests, but need not be restricted to such purposes. It expresses the ratio of agreement beyond chance to the maximum possible agreement beyond chance. A k lower than 0.11 is regarded as 'virtually no agreement' and between 0.11 and 0.40 as 'slight agreement' [9]. Kappa was calculated for raw scores, for scores categorized with the cut-offs suggested by Copes and colleagues (i.e. 1-8, 9-15, 16-24, 25-40, 41-49, 50-75) [10] and for scores in three groups, as sometimes done for reimbursement purposes (1-15, 16-24, 25-75). A comparison of the 2 scores by a Bland-Altman chart was also performed. This method, described for the first time in 1986, [11] has now become popular for comparing two methods of measurement of variables with a large number of possible values, as in our case. It plots the differences between the two scores (y axis) against the means of the two scores (x axis). Briefly, this method offers a nice 'eyeball' impression of the agreement, gives a numeric measure of the average difference and of the limits of agreement and allows one to check if the differences increase or decrease as the values of the 2 variables increase (in which case the spread is fan-like instead of horizontal).

Because the ISS derives from the sum of the squared AIS scores of the three most severe lesions of three different anatomic areas, an incomplete recording of multiple injuries (whose possible causes have been explained in the introduction) may yield a lower ISS. Therefore, we also attempted to verify if there was a relationship between the accuracy of registration of multiple injuries (i.e. the number of traumatic diagnoses for each patient) and the agreement between the 2 differently calculated ISSs. We counted both traumatic ICD-9-CM and directly assigned AIS diagnostic codes for each patient. We called DIARATIO the ratio of the number of direct AIS codes over the number of ICD-9-CM traumatic codes. Then we plotted the differences between the 2 ISSs against DIARATIO. The Kruskal-Wallis non parametric test was used to assess the significance of the absolute differences in ISS among 3 groups of patients created according to DIARATIO (< 1, 1 and > 1). To further assess the effect of the limited number of diagnoses allowed in administrative files we calculated the Cohen's kappa and Bland-Altman charts excluding all cases with more than six AIS codes.

Finally, given that the severity scoring is important for risk-adjustment, we compared the predictive power for survival (at 30 days from admission) of both scores. We evaluated two logistic regression models where survival was the dependent variable and the scores were the independent one. Discrimination was assessed by the receiver operating characteristic curve (ROC) and compared by the test for equality of two or more ROC areas [12].

## Results

Out of the 289 RITG cases, 14 had no match and 3 were burn cases that are not mapped by ICDPIC, leaving 272 cases for examination.

Table 1 displays the summary statistics of ISS scores.

**Table 1:** Summary statistics of ISS and number of diagnostic codes per patient.

	Mean	Standard deviation	Median	Min-max
Directly-calculated ISS	24	11	22	1-59
ICDPIC-calculated ISS	18	13	16	4-75
Number of AIS codes per patient	5	3	4	1-14
Number of ICD codes per patient	3	2	3	1-6

ISS = Injury Severity Score

The two scores were the same in 33/272 cases (12.1%, corresponding to a Cohen's kappa of 0.07). Table 2 shows the cross tabulation of the six ISS categories. The ISS categories were the same in 80/272 cases (29.4%, corresponding to a Cohen's kappa of 0.09). The agreement was 38.2%, kappa = 0.13, when using three groups.

**Table 2:** Cross-tabulation of the two scores grouped in six categories.

Directly calculated ISS	ICDPIC-calculated ISS						
	
	1-8	9-15	16-24	25-40	41-49	50-75	Total
1-8	**6**	1	0	1	0	0	8

9-15	8	**13**	4	2	0	0	28

16-24	19	42	**34**	13	0	2	110

25-40	8	23	33	**25**	1	3	93

41-49	1	4	7	7	**1**	1	21

50-75	1	2	5	2	1	**1**	12

Total	43	85	83	50	3	8	272

ISS = Injury Severity Score

**Figure 1:**
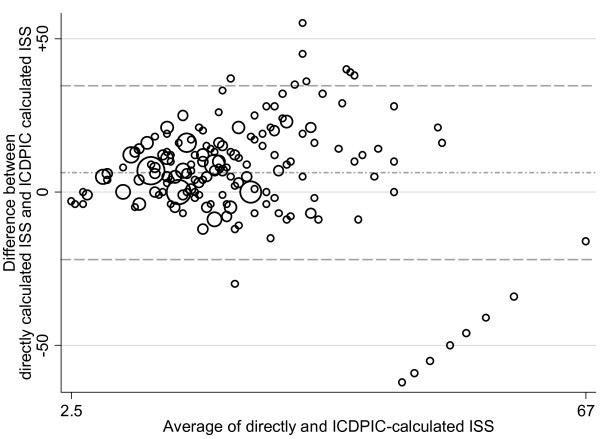
**Bland-Altman plot showing the differences between the two scores against their means**. ISS = Injury Severity Score.

Figure 1 displays the Bland-Altman plot. It can be seen that the differences are quite wide. The average difference ± standard deviation is 6.36 ± 14.45 (limits -22.0 to 34.7), confirming the general tendency of ICDPIC to underestimate severity. There is a certain degree of fan-like spread, showing that to some extent the differences increase with the average severity.

When the 61 patients with more than six AIS codes were excluded, the agreement increased negligibly: Cohen's kappa for raw scores 0.08, for six categories 0.10, for three categories 0.11, Bland Altman average difference and limits 5.01 and -21.00 to 31.02.

**Figure 2:**
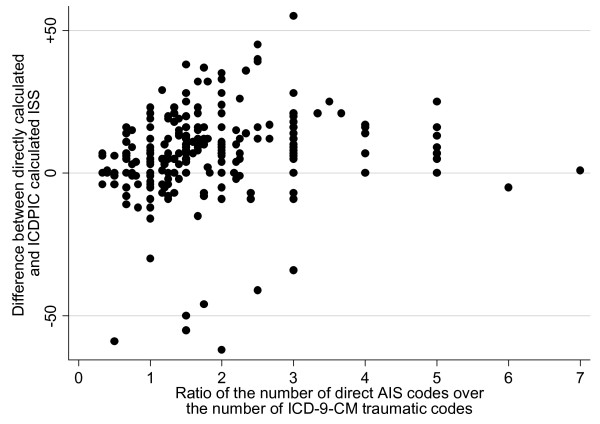
**Plot of differences between the two scores and DIARATIO**. DIARATIO = the ratio of the number of direct AIS codes over the number of ICD-9-CM traumatic codes; ISS = Injury Severity Score; AIS = Abbreviated Injury Scale.

**Figure 3:**
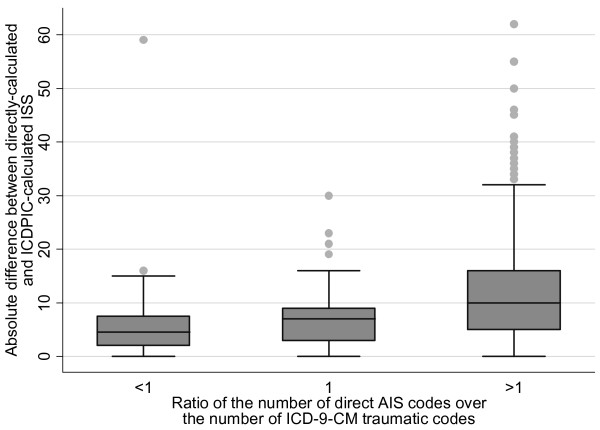
**Differences between the 2 scores in 3 groups of patients with increasing DIARATIO**. DIARATIO = the ratio of the number of direct AIS codes over the number of ICD-9-CM traumatic codes; ISS = Injury Severity Score; AIS = Abbreviated Injury Scale.

The number of diagnostic codes are shown in Table 1. In 36 cases (13.2%) there were fewer AIS than ICD-9-CM traumatic diagnostic codes, in 46 cases (16.9%) they were the same quantity, and in 190 cases (69.8%) there were more AIS diagnoses. When the quantity of directly calculated AIS codes was ≥ 6 (N = 83) there were 6 ICD-9-CM traumatic codes in only 12 (14.5%) cases and fewer than 6 in 49 (85.5%) cases. If all of ICD-9-CM codes, including the non traumatic ones, are considered, these categories become respectively 34 (41%) and 47 (59%). Figure 2 shows the plot of the differences between the 2 scores and DIARATIO. In general it seems that the differences increase when the ratio also increases. This is confirmed by the box plot in Figure 3 where these differences (absolute values) are summarised and compared among three groups of patients with increasing DIARATIO (p < 0.01).

Table 3 displays the directly calculated ISS and the ICD-9-CM diagnostic codes of the patients with an ICDPIC-calculated ISS of 75. In nearly all of them the ICD code is the same and the directly calculated ISS is much lower than 75.

**Table 3:** Patients with an ICDPIC-calculated Injury Severity Score of 75

	Directly calculated Injury Severity Score	ICD code mapped to an Abbreviated Injury Scale score of 6
Patient 1	20	862.8

Patient 2	16	862.8

Patient 3	29	862.8

Patient 4	25	862.8

Patient 5	41	862.8

Patient 6	13	806.01

Patient 7	59	862.8

Patient 8	34	862.8

**Figure 4:**
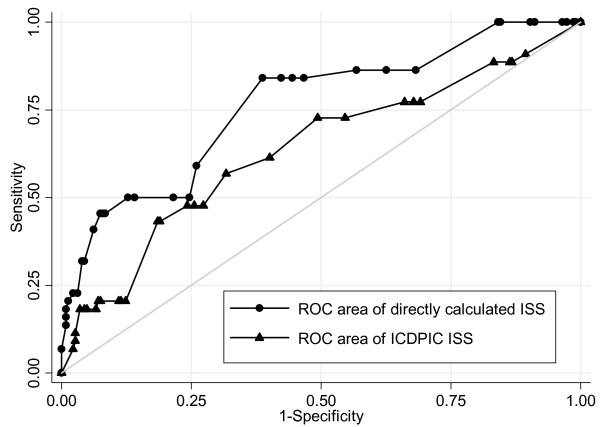
**ROC curves for prediction of survival of the 2 scores**. ROC area of directly calculated ISS: 0.7631, 95% CI 0.68-0.84. ROC area of ICDPIC ISS: 0.6355 95% CI 0.53-0.73. ISS = Injury Severity Score.

Figure 4 shows the ROC curves of the 2 ISS scores. The area under the curve of ICDPIC is significantly lower: 0.63 vs. 0.76 (p = 0.02).

## Discussion

ICDPIC is very appealing for anyone who wants to adopt AIS-based risk adjustment for injury severity, because it is inexpensive and allows exploiting the administrative data banks on hospital admissions that in many countries are comprehensive and easy to access. However, when applied to our administrative records, the ICDPIC-calculated ISS was in poor accordance with the directly calculated ISS and kappa values were invariably in the lowest range. Not even when ISS was collapsed into six or three categories, the agreement between the two methods was acceptable. The Bland-Altman chart led to the same conclusion.

There can be several reasons for this unsatisfying performance.

First of all, given that directly calculated ISS was used as the reference, a thought should be given to the quality of AIS coding. As already said, the coding was done within a research program by dedicated and expert clinicians with cross-check and external review. Its accuracy should therefore be taken for granted.

In general, trauma registries in the USA include more cases with mild injuries than European registries [13] and the case mix upon which ICDPIC was developed was probably different from ours. Then its performances would have probably been better if more patients with minor injuries - scarcely represented in our experience - had been included. However, we were not interested in evaluating ICDPIC in absolute terms, but in terms of practical usefulness in our setting, which (ISS > 15 or admission to ICU) is similar to other European trauma registries.

It must also be said that ICDPIC is meant to map ICD-9-CM codes to ISS, through AIS 1990 version, while RITG adopted the 1998 version. However, the differences between the versions are minor and unlikely to explain our findings.

Irrespective of the inherent quality of ICDPIC, its final performance depends also on the accuracy of the ICD-9-CM coding. We have shown that the number of ICD-9-CM traumatic diagnoses recorded in our ADB was lower than the number of AIS diagnoses recorded in RITG, the latter being likely closer to the real quantity. We have also shown that the differences between the two scores increase when the ratio between the number of traumatic diagnoses in RITG and in ADB also increases. This explains part of the differences in ISS. And the fact that the differences are toward an underestimation of ISS by ICDPIC, confirms it.

We have also shown that, as the agreement between the ISS scores was virtually unchanged excluding cases with more than six AIS codes, the incomplete coding in administrative charts does not depend entirely on their restriction to six ICD codes. In fact, in the majority of the cases when there were more than 6 AIS-coded injuries, still the clinicians failed to exploit the room available for an accurate coding of multiple injuries in the administrative charts. This occurred even when considering any type of diagnosis - i.e. accounting for the potential 'competition' in the ADB between traumatic and other codes. One could think that ICD-9-CM is less detailed than the AIS. The truth is just the opposite because ICD-9-CM traumatic codes are more than two thousand while there are about thirteen hundred items in AIS 1998.

There is no doubt then that in our region there is room for improving the compilation of administrative records of trauma cases, a fact that in turn would result in an improved ICD-9-CM to ISS mapping. It is difficult to say to what extent this finding can be generalized. Although many papers in the past have highlighted the inaccuracy of administrative diagnostic coding [14-17] we could not find any national or international study reporting the number of ICD-9-CM traumatic codes per record, which could allow some sort of comparison. From indirect evidence, we suspect, however, that completeness is higher the USA than in Italy and other European countries. For example, a recent American paper considering all admissions [18] reported an average of 8.6 secondary diagnoses per record, while both an Italian [19] and a Swedish [20] national surveys reported an average of about 1. Moreover, while only six ICD-9-CM codes can be assigned for each admission in Italy, up to 30 coding slots have been reported in the USA [21]. In a previous paper from the USA [22] the predictive power for mortality of ICD-mapped ISS was, as expected, worse than the one of directly calculated ISS, but the difference was much smaller than in ours, a finding that agrees well with a more complete administrative coding in the USA.

Given this suspicion, more research is warranted to quantify the possible decrease in performance of other severity scores - like ICISS and TMPM-ICD9 - developed in the USA and based on ICD-9-CM when they are applied 'in vivo' to other administrative data banks with lower completeness.

It was not our goal to assess whether ICDPIC is better of worse than its proprietary counterpart ICDMAP-90 or any other similar program, however we appreciated the effort of ICDPIC authors to make a tool of such potential usefulness freely available (at least for Stata users). Indeed, most of the mapping limitations highlighted in our setting are likely to apply to any of these programs.

We can describe though an identified weakness of ICDPIC whose correction might help to improve the program. The lower right part of the Bland-Altman chart, shows that there are 8 patients that, contrary to the general trend, have much higher ICDPIC than directly calculated ISS. A closer look at these cases (Table 3) shows that seven of these patients share the same ICD-9-CM diagnostic code 862.8 - *Injury to multiple and unspecified intrathoracic organs without open wound into cavity *- to which ICDPIC attributed an AIS 6 (which translates into ISS of 75, meaning certain death). In our opinion, this code is too generic for being invaribly mapped to AIS 6 and the contrast with the directly calculated ISS indicates that there is indeed overestimation. In the remaining case of the table, the ICDPIC calculated ISS seems reasonable (806.01 - *Closed Fracture of C1-C4 Level of Vertebral Column with Complete Lesion of Cord*) and the discrepancy with its directly-calculated counterpart is due to a mistake in ICD-9-CM coding, because the patient had a fracture without cord injury.

Finallly, the progressive diffusion of later versions of ICD and AIS (i.e. ICD 10 and AIS 2005 update 2008) should theoretically make obsolete the version of ICDPIC we investigated. However, the main problem highlighted - i.e. incomplete administrative coding - will not necessarily be solved by the adoption of ICD 10. As for AIS, it has recently been suggested to maintain parallel coding with AIS 1998 because the conversion to AIS 2005 seems problematic [23].

## Conclusions

In conclusion, the agreement between the ISS mapped by ICDPIC from 272 administrative records of major trauma patients and the one calculated prospectively by expert registrars on the same patients was poor. On average, ICDPIC tended to underestimate ISS, though in some cases it grossly overestimated it. The discriminative power of ICDPIC-ISS was significantly lower than the one of directly calculated ISS. Sub-optimal accuracy of ICD-9-CM coding, especially as concerns the complete registration of multiple injuries, is a main cause of this disagreement and its effects on other ICD-based injury severity scores are worth further research.

## Competing interests

The authors declare that they have no competing interests.

## Authors' contributions

SDB conceived the study, carried out the statistical analyses and drafted the manuscript. ST participated in the statistical analyses. FV participated in the conception of the study, participated in the statistical analysis and revised the manuscript. LZ helped to draft the manuscript. FB revised it critically for important intellectual content. All authors read and approved the final manuscript.
